# Machine learning in predicting severe acute respiratory infection outbreaks

**DOI:** 10.1590/0102-311XEN122823

**Published:** 2024-01-08

**Authors:** Amauri Duarte da Silva, Marcelo Ferreira da Costa Gomes, Tatiana Schäffer Gregianini, Leticia Garay Martins, Ana Beatriz Gorini da Veiga

**Affiliations:** 1 Universidade Federal de Ciências da Saúde de Porto Alegre, Porto Alegre, Brasil.; 2 Programa de Computação Científica, Fundação Oswaldo Cruz, Rio de Janeiro, Brasil.; 3 Centro Estadual de Vigilância em Saúde, Secretaria de Saúde do Estado do Rio Grande do Sul, Porto Alegre, Brasil.

**Keywords:** Severe Acute Respiratory Infection, Machine Learning, Computer Models, Epidemiologic Surveillance, Neural Networks (Computer), Síndrome Respiratória Aguda Grave, Aprendizado de Máquina, Modelos Computacionais, Vigilância Epidemiológica, Redes Neurais (Computação), Síndrome Respiratorio Agudo Grave, Aprendizaje Automático, Modelos de Ordenador, Vigilancia Epidemiológica, Redes Neurales (Computación)

## Abstract

Severe acute respiratory infection (SARI) outbreaks occur annually, with seasonal peaks varying among geographic regions. Case notification is important to prepare healthcare networks for patient attendance and hospitalization. Thus, health managers need adequate resource planning tools for SARI seasons. This study aims to predict SARI outbreaks based on models generated with machine learning using SARI hospitalization notification data. In this study, data from the reporting of SARI hospitalization cases in Brazil from 2013 to 2020 were used, excluding SARI cases caused by COVID-19. These data were prepared to feed a neural network configured to generate predictive models for time series. The neural network was implemented with a pipeline tool. Models were generated for the five Brazilian regions and validated for different years of SARI outbreaks. By using neural networks, it was possible to generate predictive models for SARI peaks, volume of cases per season, and for the beginning of the pre-epidemic period, with good weekly incidence correlation (R^2^ = 0.97; 95%CI: 0.95-0.98, for the 2019 season in the Southeastern Brazil). The predictive models achieved a good prediction of the volume of reported cases of SARI; accordingly, 9,936 cases were observed in 2019 in Southern Brazil, and the prediction made by the models showed a median of 9,405 (95%CI: 9,105-9,738). The identification of the period of occurrence of a SARI outbreak is possible using predictive models generated with neural networks and algorithms that employ time series.

## Resumo

Surtos de síndrome respiratória aguda grave (SRAG) ocorrem anualmente, com picos sazonais variando entre regiões geográficas. A notificação dos casos é importante para preparar as redes de atenção à saúde para o atendimento e internação dos pacientes. Portanto, os gestores de saúde precisam ter ferramentas adequadas de planejamento de recursos para as temporadas de SRAG. Este estudo tem como objetivo prever surtos de SRAG com base em modelos gerados com aprendizado de máquina usando dados de internação por SRAG. Foram incluídos dados sobre casos de hospitalização por SRAG no Brasil de 2013 a 2020, excluindo os casos causados pela COVID-19. Estes dados foram preparados para alimentar uma rede neural configurada para gerar modelos preditivos para séries temporais. A rede neural foi implementada com uma ferramenta de *pipeline*. Os modelos foram gerados para as cinco regiões brasileiras e validados para diferentes anos de surtos de SRAG. Com o uso de redes neurais, foi possível gerar modelos preditivos para picos de SRAG, volume de casos por temporada e para o início do período pré-epidêmico, com boa correlação de incidência semanal (R^2^ = 0,97; IC95%: 0,95-0,98, para a temporada de 2019 na Região Sudeste). Os modelos preditivos obtiveram uma boa previsão do volume de casos notificados de SRAG; dessa forma, foram observados 9.936 casos em 2019 na Região Sul, e a previsão feita pelos modelos mostrou uma mediana de 9.405 (IC95%: 9.105-9.738). A identificação do período de ocorrência de um surto de SRAG é possível por meio de modelos preditivos gerados com o uso de redes neurais e algoritmos que aplicam séries temporais.

## Resumen

Brotes de síndrome respiratorio agudo grave (SRAG) ocurren todos los años, con picos estacionales que varían entre regiones geográficas. La notificación de los casos es importante para preparar las redes de atención a la salud para el cuidado y hospitalización de los pacientes. Por lo tanto, los gestores de salud deben tener herramientas adecuadas de planificación de recursos para las temporadas de SRAG. Este estudio tiene el objetivo de predecir brotes de SRAG con base en modelos generados con aprendizaje automático utilizando datos de hospitalización por SRAG. Se incluyeron datos sobre casos de hospitalización por SRAG en Brasil desde 2013 hasta 2020, salvo los casos causados por la COVID-19. Se prepararon estos datos para alimentar una red neural configurada para generar modelos predictivos para series temporales. Se implementó la red neural con una herramienta de canalización. Se generaron los modelos para las cinco regiones brasileñas y se validaron para diferentes años de brotes de SRAG. Con el uso de redes neurales, se pudo generar modelos predictivos para los picos de SRAG, el volumen de casos por temporada y para el inicio del periodo pre-epidémico, con una buena correlación de incidencia semanal (R2 = 0,97; IC95%: 0,95-0,98, para la temporada de 2019 en la Región Sudeste). Los modelos predictivos tuvieron una buena predicción del volumen de casos notificados de SRAG; así, se observaron 9.936 casos en 2019 en la Región Sur, y la predicción de los modelos mostró una mediana de 9.405 (IC95%: 9.105-9.738). La identificación del periodo de ocurrencia de un brote de SRAG es posible a través de modelos predictivos generados con el uso de redes neurales y algoritmos que aplican series temporales.

## Introduction

Viral respiratory infections are easily spread in the community, affecting millions of individuals annually worldwide, representing a public health problem with high morbidity and mortality, especially in children, older adults, and immunocompromised patients ^1^. Most acute respiratory infections are caused by viruses, and symptoms, when present, range from mild (runny nose and cough) and influenza-like illnesses (ILI) to severe acute respiratory infections (SARI).

Seasonality of SARI outbreaks varies across different geographic regions ^2^. In Brazil, a large-sized country, a seasonal southward wave is observed, starting in late January in the North, and reaching the South in the middle of the year ^3^ ^,^ ^4^. Notably, intensity and duration of outbreaks also vary by region ^5^.

Influenza A (IAV) and B (IBV) viruses cause human influenza, considered one of the most important infectious diseases of humanity. Seasonal flu epidemics occur every year and, eventually, new viral subtypes with pandemic potential emerge. In 1918-1919, the humanity was ravaged by the Spanish flu, caused by IAV H1N1; in 2009, a new IAV subtype (H1N1pdm09) caused the first influenza pandemic of the 21st century ^1^. In Brazil, among the 88,464 cases of SARI hospitalization reported in 2009, 50,482 were confirmed as IAV H1N1pdm09, with 2,060 deaths ^6^.

Besides influenza virus, other respiratory viruses are also associated with SARI epidemics. In this sense, the new coronavirus (SARS-CoV-2) emerged in humans at the end of 2019, causing the COVID-19 pandemic ^7^. Within three years, more than 700 million COVID-19 cases have been reported and approximately 7 million people have died. On April 28, 2023, the World Health Organization (WHO) declared the end of COVID-19 as a public health emergency of international concern; nonetheless SARS-CoV-2 is still circulating among humans, thus constant surveillance is necessary to be prepared for outbreaks and epidemic situations ^8^.

Prevention and control of SARI outbreaks rely on constant epidemiological surveillance, and information of previous seasonal epidemics of respiratory viral infection, with accumulated data of cases, may be used to predict future epidemic seasons. Then, the development of prediction models that consider variables specific for each geographic region is important to prepare healthcare networks and to guide health authorities in decision-making for policies and planning ^9^ ^,^ ^10^. In this study, we develop and analyze predictive models for SARI outbreaks using data from Brazil. Results show that, it is possible to predict SARI outbreaks with good precision based on data from previous epidemics.

This study uses SARI notification data, but we highlight that other data sources have been used successfully in forecasting epidemics as well. For example, the U.S. Centers for Disease Control and Prevention (CDC) have actively promoted the use of predictive models to forecast influenza seasons with the usage of social media datasets ^9^.

This study aims to predict SARI outbreaks with machine learning models, using hospitalization notification data specific to SARI cases. The dataset used in this study comprises notifications of SARI-related hospitalizations in Brazil from 2013 to 2020, excluding COVID-19 cases.

## Materials and methods

### Case definition

ILI and SARI definition in Brazil is in line with that of WHO, with minor differences. According to WHO, ILI is characterized by fever > 38ºC, accompanied by cough within 10 days of infection, and SARI includes these symptoms and hospitalization of the patient ^11^. For means of epidemiological surveillance in Brazil, until 2019, case definition for ILI included fever > 38ºC, accompanied by cough or sore throat within seven days of infection; and SARI included ILI symptoms accompanied by O_2_ saturation < 95%, dyspnea, and increased respiratory rate, requiring hospitalization ^12^ ^,^ ^13^.

This study excluded data of cases that did not meet all SARI case definition, as well as duplicates. Notifications were considered duplicates when they shared the same information for the set of variables related to notification ID, municipality of notification, and notification date. Due to lack of specificity regarding self-reported fever, especially for older adults, the following criteria were adopted to filter notified cases: presence of cough or sore throat, followed by the dyspnea or O_2_ saturation < 95% or difficult breathing; with hospitalization or death. To standardize notifications over different periods, records referring to COVID-19 cases were removed.

### Data source

In Brazil, SARI cases were universally notified in 2009 in response to the H1N1pdm09 pandemic ^14^. Until 2018, notification was performed by the Brazilian Information System for Notificable Diseases (SINAN, acronym in Portuguese) ^15^, and then migrated to the Influenza Epidemiological Surveillance Information System (SIVEP-Gripe, acronym in Portuguese) ^16^, which collects data of SARI cases, including age, sex, symptoms, comorbidities, date of first symptoms, vaccination, polymerase chain reaction (PCR) results for respiratory viruses, hospitalization date, date of discharge or death, health unit and geographic location, and other data, totaling 252 variables.

In Brazil, a correlation between the occurrence of SARI and the rainy and cold periods has been observed, especially in the South ^4^ ^,^ ^5^. Considering that SIVEP-Gripe data does not include temperature as variable, and with the objective of evaluating the contribution of this variable in the generation of predictive models for SARI, the daily averages of minimum and maximum temperatures and thermal amplitude per region were added to the dataset, as well as the total average of temperatures per region. Temperature was calculated based on data from the website of the Brazilian National Institute of Meteorology (INMET, acronym in Portuguese) ^17^. The data retrieved bring values of maximum and minimum temperatures, at different times of the day in meteorological stations located in each state across the five regions.

### Data processing

For this study, the dataset returned 689,797 records of SARI cases in Brazil from January 2009 to March 2021. For the predictive models tested, the dataset was restricted to the following variables: number of daily notifications (used as a dependent variable); date of first symptoms; sex (male, female); age group (young, adult, older adult); and positive diagnosis for IAV or IBV. Fields related to SARI-related symptoms according to the surveillance protocol (fever, sore throat, dyspnea, and cough) were not used, as this would bring redundancy on the number of notifications, not giving positive effects on the generation of models.

Data were treated considering the date of the first reported SARI symptoms. Moreover, data from 2013 to 2020 were used to generate the predictive model due to the difference in protocols in the first years (2009 to 2012) and the insufficiency of data for 2021 (only three months).

After filtering and analyzing the data, the dataset containing SARI notification data was grouped by date of first symptoms, i.e., SARI notifications of the same date were summed in a single line; the grouping result showed a further reduction in the amount of data (rows of dataset available) for the generation of models.

For most variables, grouping was done by adding up the number of cases of hospitalization for SARI on each date of the first symptom that pointed to a certain category (sex, age, etc.). For sex, grouping was done in a similar way, adding up the total number of cases that pointed to the male or female categories on each date of first symptoms. Ages were grouped as young (0-19 years), adults (20-59 years), and older adults (> 60 years).

At the end of this first process of filtering and excluding records with problems, a total of 354,249 records were available for generating predictive models. The data were separated by region due to each location specificity, and, during the evaluation process, different predictive models were generated for each region. At the end of data processing, temperature data was added. These data, grouped and divided by region, were used to generate predictive models. To reduce notification bias, the 7-day moving average was applied to the number of daily notifications.

To align with the Long Short-Term Memory (LSTM) algorithm, the date of initial symptoms was deconstructed into year, month, and day components. Subsequently, the values within each category were normalized to a scale ranging from 0 to 1. As part of streamlining the predictive model generation process, the analysis includes weeks of the year instead of epidemiological weeks. This simplification facilitates a more straightforward and coherent framework for the development and interpretation of the predictive models.

### Predictive models

A range of machine learning algorithms were tested to model the observed seasons in each region to predict the next. Since this study deals with multivariate time series ^18^ with seasonality, the LSTM recurrent neural network (RNN) was chosen ^19^. Data were separated into two sets: 80% for model training and 20% for model testing. The dataset used for training and test used data available from the years prior to the year in which the prediction is to be made, with 2013 as the beginning of the period. Test data (20%) corresponds to the most recent period of the data set. Supplementary Material - Table S1 (https://cadernos.ensp.fiocruz.br/static//arquivo/suppl-e00122823_2613.pdf) presents parameters of the neural network, which was trained using the Knime tool and yielded the highest performance.

### Tools

The Knime (https://www.knime.com/), a pipeline-based tool, was employed for data preparation, machine learning model generation, and model validation, thereby generating the results for analysis. For result analysis, the programming language R (http://www.r-project.org) and the RStudio interface (https://rstudio.com/) were employed. The Knime workflow, the R code of the web tool for visualization, and user instructions are available on GitHub (https://github.com/vigilanciaepidemiologica/sari_prediction).

## Results

### Generation of predictive models

Although the original dataset contains data from the five Brazilian regions (North, Northeast, Central-West, Southeast, and South), the analyses were preferably conducted with data from the South region due to higher consistency and a better-defined period of SARI occurrence (winter months) in relation to the other regions. We noted a significant variation on volume of annual notifications of SARI hospitalization. For example, in the South Region, 2014 and 2015 had low volumes of notifications (4,754 and 4,572, respectively) and, conversely, 2016 and 2020 had large volumes of notifications (11,266 and 22,140, respectively). Table 1 shows the volume of SARI notifications by region. The total number of records corresponds to the total number of SARI notifications and the total number of treated records corresponds to notifications grouped by day (data from 2013 to 2020).

**Table 1:** Amount of severe acute respiratory infection (SARI) data for each Brazilian region (2013 to 2020).

Region	Total records (1)	Total records treated	Total records in 2020 (2)	Percentage (%) of records (2/1)
North	22,994	2,361	13,129	57.10
Northeast	60,908	2,837	36,335	59.66
Central-West	29,712	2,653	12,660	42.61
Southeast	164,354	2,897	83,763	50.96
South	76,281	2,888	22,140	29.02
Total	354,249	13,636	168,027	-

Table 2 shows the test results for R^2^ to emphasize the importance of the volume of data for the generation of SARI predictive models. As the period decreases and, consequently, the amount of data for model generation also decreases, R^2^ assumes smaller values, indicating that the model will not provide a good prediction with smaller time windows.

**Table 2:** Comparison of the volume of data available in each region and period, with training results using the R^2^ metric.

Region	2013-2018		2014-2018		2015-2018		2016-2018		Notifications (2013-2018)
	Total records	R^2^	Total records	R^2^	Total records	R^2^	Total records	R^2^	
Southeast	2,173	0.96	1,811	0.73	1,453	0.44	1,094	0.05	98,166
South	2,164	0.98	1,810	0.97	1,452	0.90	1,093	0.80	48,942
Northeast	2,114	0.95	1,763	0.77	1,417	0.43	1,082	0.02	42,892
Central-West	1,929	0.67	1,618	0.45	1,329	0.41	1,046	0.10	17,604
North	1,644	0.58	1,403	0.33	1,176	0.60	1,000	0.00	16,696

Note: the evaluation metric of the predictive models is compared with the number of severe acute respiratory infection (SARI) notification records by each region of Brazil. For R^2^, validation is being considered for the volume of data from 2013 to 2018.

The generation of predictive models was simulated using machine learning algorithms such as Random Forest, Naive Bayes, Tree Assemble, and the Resilient Backpropagation (RPROP) Multi-Layer Perceptron (MLP); however, the verified accuracies were very low. Given the nature of the problem to be solved, a time series approach was shown to be more appropriate. Therefore, we used LSTM to generate predictions, which can perform well with seasonal time series ^19^.

In a comparison, the application of LSTM had better accuracy than the use of the Seasonal Autoregressive Integrated Moving Average (SARIMA) method. As Table 3 shows, the model generated with LSTM had a better metric in the model training step for almost all periods, except for the period with the least amount of data. The data used are from SARI notification for the South Region.

**Table 3:** Comparison of the Seasonal Autoregressive Integrated Moving Average (SARIMA) method and neural network models with Long Short-Term Memory (LSTM) using the R^2^ metric.

	2013-2018	2014-2018	2015-2018	2016-2018	2017-2018	2018-2018
Records	2,164	1,810	1,452	1,093	730	365
SARIMA (R^2^)	0.66	0.68	0.68	0.65	0.54	0.46
Neural network (R^2^)	0.98	0.97	0.90	0.80	0.72	0.04

Then, different categories (independent variables) were used to investigate models with better performance and, therefore, different metrics were obtained for the tests, with the categories sex (male and female), age group (young, adult, and older adult), and positive diagnosis for IAV and IBV. These categories showed the best performance in the prediction made in the test stage, with determination coefficient (R^2^) = 0.99; mean absolute error (MAE) = 1.28; mean square error (MSE) = 2.89; root mean square error (RMSE) = 1.70; mean squared deviation (MSD) = 0.39; and mean absolute percentage error (MAPE) = 0.06. Notably, no significant improvements in accuracy were found when using temperature variables to generate the predictive models. Therefore, this variable was not used in the models presented as results in this study. Supplementary Material - Table S2 (https://cadernos.ensp.fiocruz.br/static//arquivo/suppl-e00122823_2613.pdf) presents metrics for other combinations of categories.

### Validation

For validation, models were generated to predict the reporting curves of SARI cases in years for which data are already available. The approximation of the curves was verified, considering the week of the beginning of the pre-epidemic period, volume of cases of notification in the season, and peak week. Accuracy metrics were used in the comparison (Table 4). To generate these metrics, 100 simulations were created for each scenario tested. We report metrics in median and 95% confidence interval (95%CI). Due to the COVID-19 pandemic, which started in 2020 and remained active until 2022, most validations were made for 2019, which, in addition to not showing the pandemic bias, also presents the largest amount of data for training models, as it accumulates data from previous years, starting in 2013.

**Table 4:** Values of prediction validation metrics, using the median and 95% confidence interval (95%CI), considering the time series of weekly cases for the South and Southeast regions, Brazil (2018 and 2019).

Target year/region	Pearson Median (95%CI)	R^2^ Median (95%CI)	RMSE Median (95%CI)	MAPE Median (95%CI)	Observed cases	Predicted cases (95%CI)
2019 - South	0.90 (0.90-0.91)	0.82 (0.81-0.83)	52 (51-53)	11 (10-14)	9,936	9,405 (9,105-9,738)
2018 - South	0.84 (0.81-0.86)	0.71 (0.66-0.73)	87 (81-94)	19 (17-24)	8,918	6,906 (6,348-7,550)
2019 - Southeast	0.98 (0.97-0.99)	0.97 (0.95-0.98)	35 (30-46)	10 (7-13)	14,332	14,399 (13,763-15,022)
2018 - Southeast	0.80 (0.75-0.83)	0.63 (0.56-0.69)	147 (141-155)	28 (24-32)	13,390	10,581 (9,616-11,503)

MAPE: mean absolute percentage error; RMSE: root mean square error.

Note: the last two columns show the annual (seasonal) volume of reported severe acute respiratory infection (SARI) cases, observed and predicted, respectively.

### Model application

Using time series algorithms, the prediction models generated with neural networks showed good results in predicting SARI outbreak seasons. As shown in Table 4, the generated models had good performance in the prediction. For both the South and Southeast regions, the performance measures were better for 2019 due to the greater amount of data for training the models. This relationship between volume of data and performance of the models was observed in the simulations for previous years, as the farther away from the present time, the less data is available for training.

**Figure 1:**
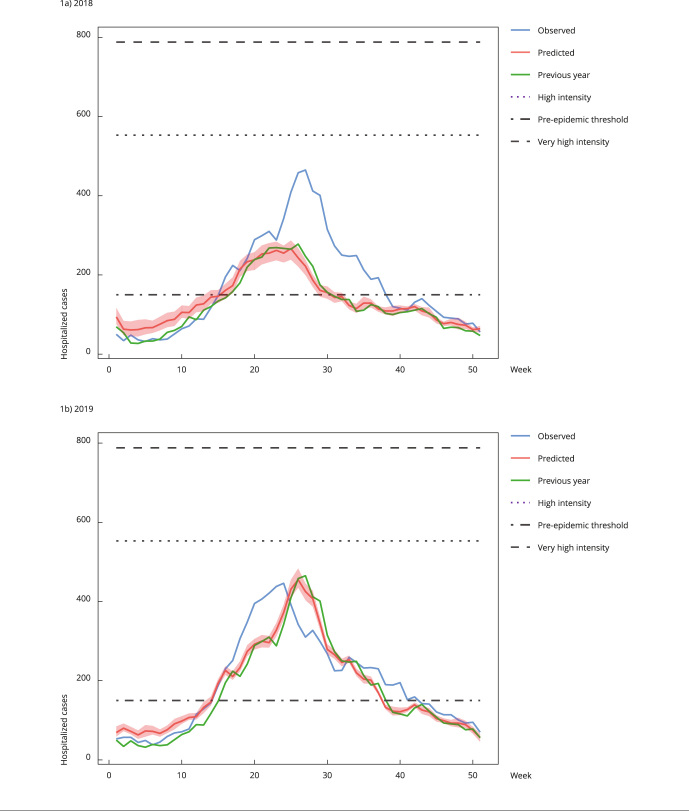
Prediction of hospitalized severe acute respiratory infection (SARI) cases for the South Region, Brazil (2018 and 2019).

Note: the graphs show the curves of cases observed in the previous year (green), cases observed in the year (blue), and the median of the prediction of cases (red) with their variability. It also identifies the volumes of cases that characterize the pre-epidemic period, with high intensity and very high intensity.

**Figure 2:**
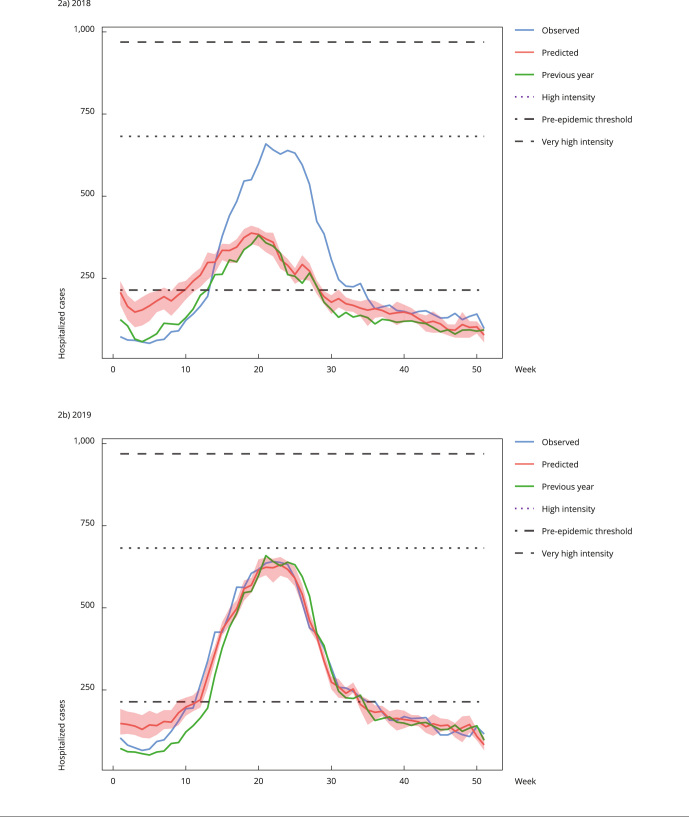
Prediction of hospitalized severe acute respiratory infection (SARI) cases for the Southeast Region, Brazil (2018 and 2019).

Note: the graphs show the curves of cases observed in the previous year (green), cases observed in the year (blue), and the median of the prediction of cases (red) with their variability. It also identifies the volumes of cases that characterize the pre-epidemic period, with high intensity and very high intensity.

Figures 1 and 2 show prediction curves generated by the models. As already mentioned, curves generated for 2019 showed greater accuracy with the observed curve. The previous year curve (green) is shown to assess whether the simple application of data observed in the previous year would be a way of predicting the following year. The variability of the generated models, obtained from random seeds, is small, with lower and upper limits very close to the median. The metrics collected for this graph showed a good performance prediction, presenting a Pearson’s coefficient of 0.90 (95%CI: 0.90-0.91), R^2^ = 0.82 (95%CI: 0.81-0.83), RMSE = 52 (95%CI: 51-53), and MAPE = 11 (95%CI: 10-14).

**Figure 3:**
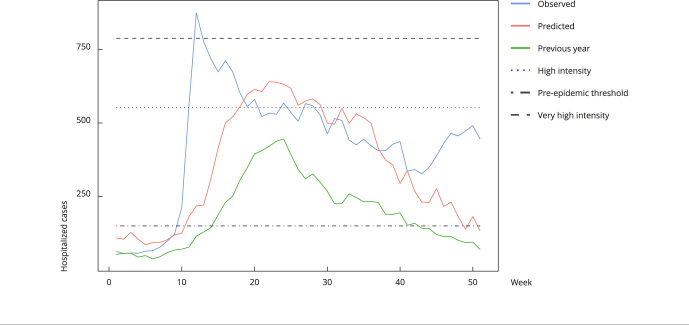
Prediction of hospitalized severe acute respiratory infection (SARI) cases for the South Region, Brazil (2020).

Note: the graphs show the curve of SARI hospitalization cases reported in the previous year (green), curve of data observed in the year (blue), and prediction curve of number of cases (red).

There is an approximation of the predicted curve of a year (red lines in Figures 1 and 2) and the curve of cases in the previous year (green lines in Figures 1 and 2). However, this approximation is not always observed. In fact, as shown in Figure 3, the generated model shows a clear difference between the predicted curve for 2020 and the curve of 2019. Noteworthy, the volume of cases predicted for 2020 (18,078) offered a very good approximation to the volume of cases observed (21,860; 17% difference), despite the low number of cases notified in 2019 (9,901 cases, 55% difference). This is an interesting result considering that 2020 was an atypical year, during which the COVID-19 pandemic brought problems such as inconsistent notifications, especially in the initial peak. Hence, the simple use of the previous year notification curve is not an effective way to predict notification of SARI hospitalization cases for the following year.

**Figure 4:**
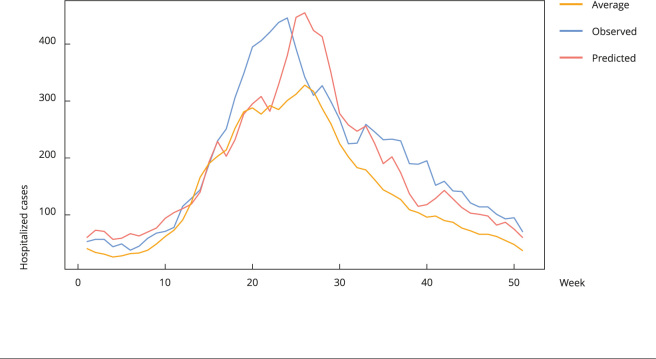
Prediction of hospitalized severe acute respiratory infection (SARI) cases for the South Region, Brazil (2019).

Note: the graph shows the curve of the average number of hospitalized cases notified in the period from 2013 to 2018 (yellow) and the curve of the data observed in 2019 (blue).

Another comparative approach was to use the average number of notifications on hospitalized SARI cases from 2013 to 2018 for the South region. An average volume of 7,357 notifications was obtained against 9,936 observed and 9,327 notification cases predicted by the model (Figure 4). It is important to note that the peak of notifications of the predicted curve approximates the peak of the mean over previous seasons (week 26), and both are two weeks apart from the observed curve.

## Discussion

In this work, we sought to find predictive models for SARI outbreaks using machine learning that present good performance (shown through metrics). These models can help guide adequate public health policies, in addition to allocating human and material resources in the necessary quantities and in a timely manner.

As results show, a prediction with good performance is possible. However, exceptional events, such as emergence of new viruses and pandemics, can be unfavorable to the prediction of models. Nevertheless, these models provide important information for healthcare management.

Our study also shows that the quality and quantity of data influence the performance of predictive models. As for quality, it is noted that the South and Southeast regions - which have a more uniform and concise notification - have a better the performance of predictive models than for other Brazilian regions. The number of notifications is impacted by the region’s population, but we noted that timely notifications also impact the volume of data available for the generation of predictive models. The South Region is a typical case where SARI notifications are made timelier ^20^.

We highlight that, except for the South Region, Brazil had a concentration of records in 2020 due to the COVID-19 pandemic. This factor influenced the performance of models, in addition to the total number of records for each region. One explanation for this is the fact that respiratory infections are more common in regions with colder winters, such as the South Region ^3^ ^,^ ^4^, but SARS-CoV-2 reached all geographic regions significantly; therefore, the increase in case notification in relation to previous years was relatively greater in the other regions. In addition, it is possible that the alert situation during the pandemic led health institutions to adopt a more strict SARI notification.

Obtaining a metric with great value (R^2^ = 0.998) in the model training test does not indicate that the prediction performance will be similar when using real data, but it is an indicator that the prediction will be very close to the observed situation. As shown in Figure 1, for 2019 the model was able to predict the beginning of the pre-epidemic threshold with good accuracy. The volume of notifications in 2019 was 9,936 cases and the expected volume was 9,327 cases (-6%). If we had used the notification curve for 2018 to predict 2019, we would have 8,918 cases (-10%) and the pre-epidemic threshold would be off by one week.

In the decomposition of a historical series of SARI notifications, using moving averages, for the South region in 2019, we can observe that the seasonal factor has an impact on the notification curve. Additionally, the presence of noise shows significant variations, hampering the approximation of a prediction curve. Even so, a predictive model based on neural networks and time series can absorb these difficulties (Supplementary Material - Figure S1; https://cadernos.ensp.fiocruz.br/static//arquivo/suppl-e00122823_2613.pdf).

The use of time series analysis, specifically LSTM, was evident in a relevant study focused on predicting the trajectory and potential cessation period of the COVID-19 pandemic in Canada ^21^. The employment of the LSTM model in this study yielded a remarkable short-term accuracy rate of 93.4%, while achieving a long-term forecast accuracy of 92.67%. These good results stress the efficacy of LSTM-based time series analysis in accurately capturing and predicting the dynamics of the COVID-19 pandemic.

The main limitation of this study is related to the quality and quantity of data. With the need to standardize data collection, it was possible to use only data from years 2013 to 2020, given that previous years used other data collection and recording standards. Some regions of Brazil present fewer registered cases annually, so the number of available records was not favorable to the generation of better models, especially for regions with fewer records. Although SARI notification, particularly influenza, is mandatory, it is not possible to guarantee that all health care units did, in fact, notify all suspected and confirmed cases each season, especially before 2020. This can lead to a subnotification of cases in each state and region. It is reasonable to assume that this effect can be greater in those regions that were historically less impacted by SARI (North and Northeast). Nonetheless, if the notification effort is sufficiently homogeneous over time in each state, this bias can be regarded as systematic. Therefore, it should not impact models accuracy regarding notified data.

On the other hand, periods with sudden changes in the volume of notifications, such as 2016 - when high numbers of influenza cases were reported due to a new H1N1pdm09 strain ^22^ - and 2020, with the COVID-19 pandemic. Even employing state-of-the-art models, accurately predicting sudden and significant shifts, such as the emergence of COVID-19, remains an inherently challenging task ^23^. Finally, the COVID-19 pandemic caused a significant impact in the laboratorial response capacity due to the high number of SARS-CoV-2 suspected cases to be tested. This challenge may have affected the capacity to test SARI cases for other respiratory viruses of interest such as the influenza viruses. If so, the number of influenza positive SARI cases notified could have been an underestimate to the real scenario in 2020. Despite these inherent limitations, it is important to acknowledge that the notification system in Brazil can be regarded as highly effective.

## Conclusion

Predictive models generated based on neural networks and algorithms that apply time series can identify the period of occurrence of a SARI outbreak. The prediction offered by these models can help public administrators define strategies to mitigate and combat outbreaks, contributing to the safety and quality of the population health, and avoiding unnecessary expenditure on human and financial resources. For instance, estimates for the expected volume of SARI notifications, the beginning of the pre-epidemic threshold, and the peak week of notifications can be of great value in defining public health policies.

Abnormal events such as pandemics, and the reduced volume of available data are factors that hinder predictive model generation. These models tend to present increasing quality due to accumulation of annual data, which will allow generation of more assertive models.

Even though it is not possible to generate prediction models that perfectly fit the observed curves, the performance metrics obtained in this study are very favorable and show the main points for decision making, such as peak week of an outbreak, case volume, and pre-epidemic threshold.
